# Sodium silicate promotes wound healing by inducing the deposition of suberin polyphenolic and lignin in potato tubers

**DOI:** 10.3389/fpls.2022.942022

**Published:** 2022-08-25

**Authors:** Ye Han, Ruirui Yang, Qihui Wang, Bin Wang, Dov Prusky

**Affiliations:** ^1^State Key Laboratory of Aridland Crop Science, Gansu Agricultural University, Lanzhou, China; ^2^College of Food Science and Engineering, Gansu Agricultural University, Lanzhou, China; ^3^Department of Postharvest Science of Fresh Produce, Agricultural Research Organization, Rishon LeZion, Israel

**Keywords:** wound healing, sodium silicate, potato tubers, suberin, lignin

## Abstract

Wound healing is a postharvest characteristic of potato tubers through accumulating suberin and lignin, which could reduce decay and water loss during storage. This study aimed to explore the impact and mechanisms of sodium silicate on wound healing of potatoes. After being wounded, “Atlantic” potato tubers were treated with water or 50 mM sodium silicate. The results showed that sodium silicate treatment accelerated the formation of wound healing structures and significantly reduced the weight loss and disease index of tubers. Furthermore, sodium silicate induced the genes expression and enzyme activity of phenylalanine ammonia lyase (PAL), 4-coumarate: coenzyme A ligase (4CL), and cinnamyl alcohol dehydrogenase (CAD) involved in the phenylpropane metabolism, enhancing the synthesis of the main precursors of suberin polyphenolic (SPP) and lignin, such as coniferyl alcohol, sinapyl alcohol, and cinnamyl alcohol. Meanwhile, the gene expression of *StPOD* and *StNOX* was activated, and the production of O^2−^ and H_2_O_2_ was promoted, which could be used for injury signal transmission and oxidative crosslinking of SPP monomers and lignin precursors. Besides, antimicrobial compounds, total phenolics, and flavonoids were also induced. We suggest that sodium silicate could promote wound healing by inducing the deposition of SPP, lignin, and antimicrobial compounds in potato tubers.

## Introduction

Potato tubers (*Solanum tuberosum* L.) are vulnerable to injury during mechanized harvesting and commercial processing, resulting in a high postharvest decay rate (Wahrenburg et al., 2021). Interestingly, many kinds of fruits and vegetables have the ability of automatic healing through accumulating suberin and lignin, especially potato tubers (Zheng et al., 2020; Wei et al., 2021). It has been reported that suberin is divided into suberin polyphenolic (SPP) and suberin polyaliphatic (SPA) (Sabba and Lulai, 2002; Lulai and Neubauer, 2014). However, the formation of a complete healing structure usually needs more than 2 weeks (Lulai and Neubauer, 2014), which is not conducive to postharvest application. Therefore, it is necessary to explore the factors that affect the rate of wound healing to accelerate its formation. Many factors had been reported to affect the formation rate of healing structure, including varieties (Wahrenburg et al., 2021), maturity (Kumar et al., 2010), environmental factors such as light (Tanios et al., 2020), temperature (Yang et al., 2020), oxygen concentration (Wei et al., 2018), humidity, and hormones (Lulai and Suttle, 2004; Zhou et al., 2019).

Silicon (Si) is considered to be one of the most beneficial mineral elements for plants (Al Murad et al., 2020). Importantly, Si has been widely reported in improving plant resistance to biotic stresses. For example, Si can improve the resistance of cucumber, wheat, and grape to powdery mildew, as well as the resistance of rice to blast and sheath blight (Ranjan et al., 2021). The mechanisms by which Si enhances plant resistance to biotic stresses are diversified and multilayered. For example, Si can be directly deposited in plant cells to enhance the strength of the cell wall, which could provide a barrier to prevent pathogen invasion (Alhousari and Greger, 2018). Si can also enhance the defense-related metabolism of plants to generate more antimicrobial substances, such as lignin and phenolics, and more defense-related enzymes, like peroxidase (POD) (Ahanger et al., 2020). Furthermore, Si is dependent on the interaction with other hormones to improve plant resistance to pathogens (Ghareeb et al., 2011).

As an abundant element in soil, Si also has a beneficial effect on plants by improving their resistance to abiotic stresses. For example, Si supplementation could improve the salt tolerance of plants by improving growth, photosynthesis, antioxidant defense systems, and hormone levels (Arif et al., 2021). Si can reduce cuticle water loss and improve rice resistance to drought stress by directly depositing on the leaf epidermis (Abed-Ashtiani et al., 2012). Si treatment alleviated heat stress in plants by enhancing abscisic acid (ABA) and salicylate (SA) hormone signaling (Khan et al., 2020). Si could increase plant resistance to heavy metals by enhancing the contents of phenolics, such as catechins, quercetin, and flavonoids (Kidd et al., 2001). In a word, the role of Si in improving plant resistance to biotic and abiotic stresses has been widely reported. However, the effects of sodium silicate on the wound healing of potato tubers and how it is achieved have not been reported.

In this study, “Atlantic” potato tubers were wounded and treated with sodium silicate or water. During healing, the weight loss rate and the disease index were measured, and the accumulation of SPP and lignin was observed. Furthermore, the gene expression and enzyme activity in phenylpropane metabolism, as well as the content of lignin precursors and lignin were measured. Meanwhile, the gene expression of NADPH oxidases (NOX) and POD, and the content of ${\text{O}}_{2}^{-}$ and H_2_O_2_ were analyzed. The contents of total phenolics and flavonoids were also investigated.

## Materials and methods

### Plant materials and treatment

Potato (*Solanum tuberosum* L. cv. Atlantic) tubers were gained from a commercial farm in Dingxi City, Gansu Province, China, in April 2019. The tubers with uniform size, no disease, and no mechanical damage were selected. After cleaning and disinfecting with sodium hypochlorite, the tubers were randomly divided into two groups. Three biological replicates per group were made in all experiments, and each replicate contained 180 tubers.

All tubers were first cut in half to simulate damage, then one group was immersed in 50 mM sodium silicate solution for 3 min (Si group), and the other was immersed in distilled water for 3 min (CK group). The concentration of sodium silicate solution was determined by a preliminary experiment (data not shown). After the surface moisture was dried naturally in the air, the tubers were put into a perforated plastic bag (30 cm × 40 cm and 0.02 mm thick) with 10 tubers per bag and stored in the dark, at normal temperature (20–25°C) and 85% humidity for wound healing. Healing tissues with a surface thickness of 2 mm were collected on 0, 1, 3, 5, 7, and 14 days, quick-frozen with liquid nitrogen, and stored at −80°C.

### Determination of weight loss rate and disease index

The weight loss rate and disease index were determined according to Yang et al. (2020). The weight loss rate was obtained by measuring the change in the weight of the tubers. It was calculated from the percentage difference between the weight of the tubers at the determined time and the initial weight. To obtain the disease index, the spore suspension of *Fusarium sulphureum* was applied to the wound site of tubers. After 7 days, the disease index was calculated according to the following equation:


$$\begin{array}{l} \text{Disease index = Σ}\;(\text{the number of diseased wounds}\\ \;\times \text{ the level of disease})\text{/4 }\times \text{ total wounds }\times \;100\%. \end{array}$$

The level of disease: 0 = no diseased area, 1 = the diseased area covering 1/4 of the wound area, 2 = the diseased area covering 1/2 of the wound area, 3 = the diseased area covering 3/4 of the wound area, and 4 = the diseased area covering the total wound area.

### Microscopic observation of SPP and lignin

According to the method of Yang et al. (2020), the accumulation of SPP was observed by spontaneous fluorescence, and the accumulation of lignin was observed by phloroglucinol staining. The thickness of the cell layers was measured by IS Capture software.

### Analysis of genes expression levels

The total RNA and cDNA were extracted by RNA Kit and Fast King RT Kit (TianGen Biotech, Beijing, China). SYBR Kit (TaKaRa, Dalian, China) and Light Cycler 96 (Roche, Switzerland) were used for qPCR. The PCR conditions were as follows: initial heating for 3 min at 95°C, followed by 40 cycles of 10 s at 95°C, 30 s at 55°C, and 20 s at 72°C. The comparative C_T_ (2^−ΔΔCT^) method was used for data calculation (Livak and Schmittgen, 2001). Primer sequences used are listed in Table 1, with *StEF1*α as an internal reference gene.

**Table 1:** Primer sequences used in this study.

**Gene name**	**Gene number**	**Primer sequences (5^′^−3^′^)**
*StEF1α*	Soltu.DM.06G005580.1	F: ATTGATGCCCCTGGTCACAG R: CATGTTCACGGGTCTGACCA
*StPAL*	Soltu.DM.09G005700.1	F: ATGGCTTCTTACTGCTCG R: GGCTACTTGGCTTACGGT
*St4CL*	Soltu.DM.03G032090.1	F: GTGTTTGCGTTTATTGGC R: GCGTAGTCCTTCACTTTCC
*StCAD*	Soltu.DM.01G046930.1	F: AAGCTGCTGATTCACTT R: GATGCTCTTTCTCCCTA
*StNOX*	Soltu.DM.03G032210.1	F: CGGAATCTACTGACATCGG R: CAGCCACAGAGTCTTCACG
*StPOD*	Soltu.DM.06G010770.1	F: AGGGACTGCTCCATTCTG R: CGGTTATCACCCATCTTA

### Determination of enzyme activity

The enzyme activity of phenylalanine ammonia lyase (PAL), 4-coumarate: coenzyme A ligase (4CL), and cinnamyl alcohol dehydrogenase (CAD) was determined according to Han et al. (2022). Simply, after extracting enzyme from 2.0 g samples, substrates L-phenylalanine, p-coumaric acid, and trans-cinnamic acid were added to measure PAL, 4CL, and CAD activities, respectively. For PAL, the extraction buffer consisted of 0.1 M borate buffer (5 ml, pH 8.8) containing 40 g/L PVP, 2 mM EDTA, and 5 mM β-mercaptoethanol. The reaction buffer consisted of 50 mM borate buffer (3 ml, pH 8.8) containing 20 mM L-phenylalanine (0.5 ml). For 4CL, the extraction buffer consisted of 0.15 M Tris-HCl buffer (3 ml, pH 8.0) containing 25% glycerol and 100 mM dithiothreitol. The reaction buffer consisted of 15 μM MgCl_2_ (0.5 ml), 5 μM p-coumaric acid (0.2 ml), 50 μM ATP (0.15 ml), and 1 μM coenzyme (0.15 ml). For CAD, the extraction buffer consisted of 0.1 mM phosphoric acid buffer (3 ml, pH 6.25) containing 10 mM β-mercaptoethanol, 2% (w/v) polyethyleneglycol, and 40 g/L PVP. The reaction buffer consisted of 10 mM NADP and 5 mM trans-cinnamic acid.

After the reaction for 1 h, the absorbance of the solution was determined at 290 nm, 333 nm, and 340 nm, respectively; 0.01 increase of absorbance per min was defined as one unit (U) of PAL activity; 0.01 increase of absorbance per hour was defined as one unit of 4CL activity; and 0.001 increase of absorbance per min was defined as one unit of CAD activity. All three enzyme activities were expressed as U/mg Prot.

### Determination of lignin precursors and lignin content

The content of lignin precursors was measured according to Ayaz et al. (2005). Lignin precursors were extracted with 70% ethanol under ultrasound. The maximum absorbances of coniferyl alcohol, sinapyl alcohol, and cinnamyl alcohol were 263 nm, 273 nm, and 273 nm, respectively. The content of lignin precursors was expressed as μg/kg FW.

The content of lignin was measured according to Yin et al. (2010). Samples (5 g) were ground into homogenate in 5 ml 95% ethanol. The precipitate was washed with 95% ethanol, ethanol, and n-hexane (1:2, V/V) solution 3 times. After drying, 1 ml of 25% acetyl glacial acetic acid bromide was added to the precipitate and reacted at 70°C for 30 min. Then, 2 ml glacial acetic acid and 0.1 ml of 7.5 M hydroxylamine hydrochloric acid were added. After centrifugation, 0.5 mL supernatant was used and 4.5 ml glacial acetic acid was added. The OD value of the solution was determined at 280 nm. The content of lignin was expressed as OD 280/g FW.

### Determination of ${\text{O}}_{2}^{\text{–}}$ and H_2_O_2_ content

The content of ${\text{O}}_{2}^{-}$ and H_2_O_2_ was measured using ${\text{O}}_{2}^{-}$ Content Test Kit and H_2_O_2_ Assay Kit (Jian Cheng Bioengineering Institute, Nanjing, China), respectively; 0.5 g of sample was added with 4.5 ml physiological saline and ground into homogenate. After incubation on ice for 2 h, the mixture was centrifuged at 8,000 *g* for 30 min, and the supernatant was used. The corresponding reagent was added according to the manufacturer's instructions, and ${\text{O}}_{2}^{-}$ and H_2_O_2_ contents were determined by measuring the OD at 550 nm and 405 nm, respectively. Both ${\text{O}}_{2}^{-}$ and H_2_O_2_ contents were expressed as mmol/g Prot.

### Determination of total phenolics and flavonoids content

The determination of total phenolics and flavonoids was based on Jiang et al. (2019) and partially modified; 6 ml of 0.5% acetic acid containing 70% acetone solution was added into 1 g tissue powder and extracted at 4°C for 24 h. The supernatant was obtained by centrifugation at 8,000 *g* for 30 min. For the determination of total phenolics, the extraction buffer consisted of 0.5 ml supernatant, 2.5 ml diluted Folin-Ciocalteu's phenol reagent (1:10), and 2 ml of 7.5% Na_2_CO_3_, and the OD value was measured at 760 nm. For the determination of flavonoids, the extraction buffer consisted of 0.5 ml supernatant, 0.15 ml of 10% AlCl_3_·6H_2_O, and 0.15 ml of 0.5% NaNO_2_, and the OD value was measured at 510 nm. The contents of total phenolics and flavonoids were expressed as gallic acid equivalents (GAE) mg /10^−1^ g FW and equivalents (CE) mg /10^−1^ g FW, respectively.

### Statistical analysis

Data were evaluated by ANOVA using SPSS Statistics 22.0. Fisher's least significant difference (LSD) test was used, and *P*-values below 0.05 were considered statistically significant. Data on graphs were shown as the mean value ± standard error. All experiments had three biological replicates.

## Results

### Sodium silicate treatment enhanced the efficacy of wound healing

**Figure 1:**
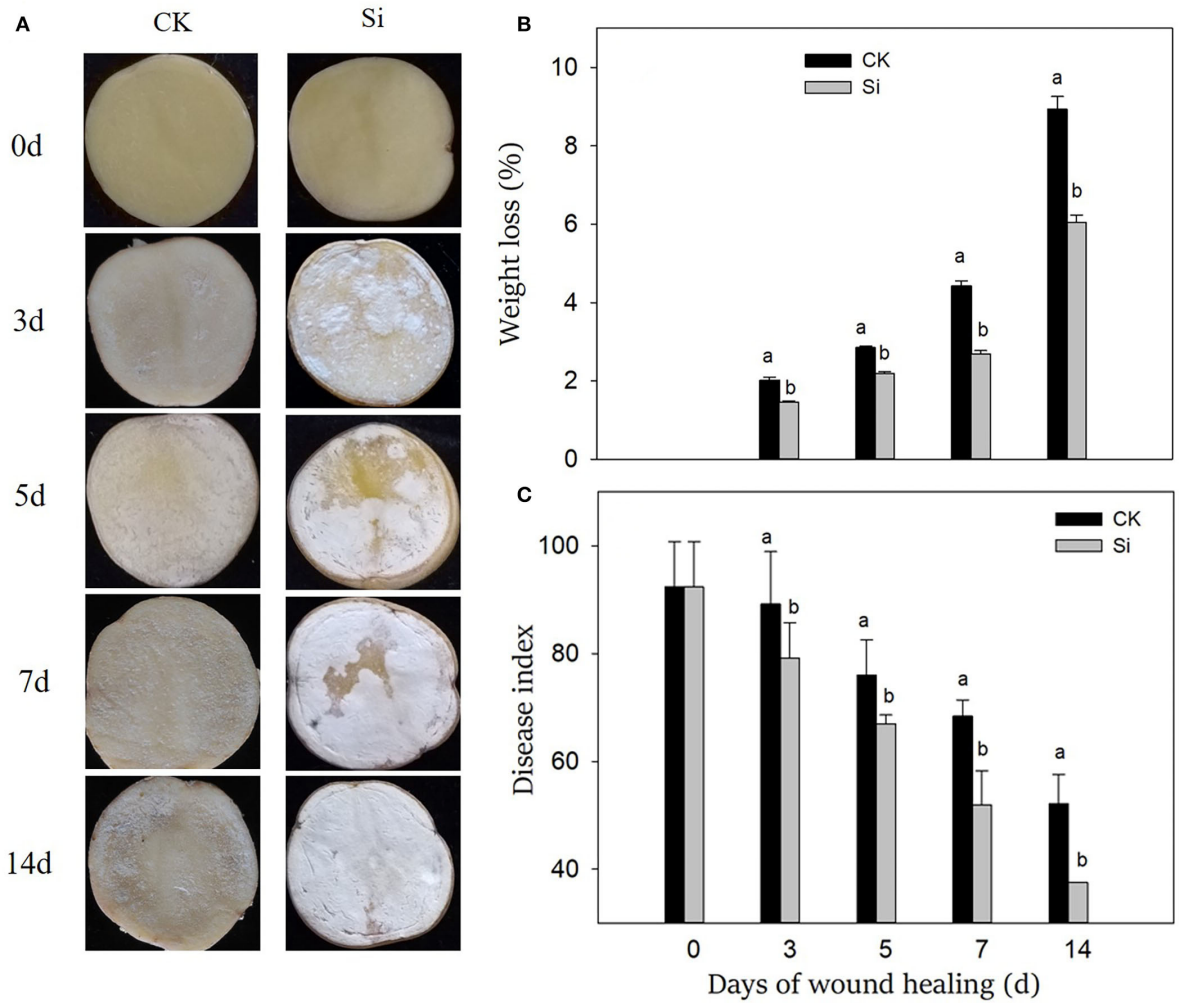
Effect of sodium silicate treatment on wound appearance **(A)**, weight loss **(B)**, and disease index **(C)** of potato tubers during healing. Vertical bars indicate the standard error of three replicate assays. Columns with different letters at each time point are significantly different (LSD, *P* < *0.05*).

After the tubers were wounded, the healing structure on the wound surface gradually thickened, which was more obvious in the Si group than that in the CK group (Figure 1A). After 3 days of wound healing, a large number of visible healing structures had accumulated on the surface of the wound in the Si group, and these white structures gradually covered the wound surface. At the end, the surface of the wound was completely covered by an obvious healing structure, whereas the tubers in the CK group had significantly less of this healing structure.

During wound healing, the weight loss rate increased gradually, and the disease index decreased gradually (Figures 1B,C). Both the weight loss rate and disease index in the Si group was always lower than that in the CK group. On 14th day of healing, the weight loss rate of tubers in the Si group was 32% lower than that in CK, and the disease index was 28% lower than that in the CK group.

### Sodium silicate treatment promoted the deposition of SPP and lignin

**Figure 2:**
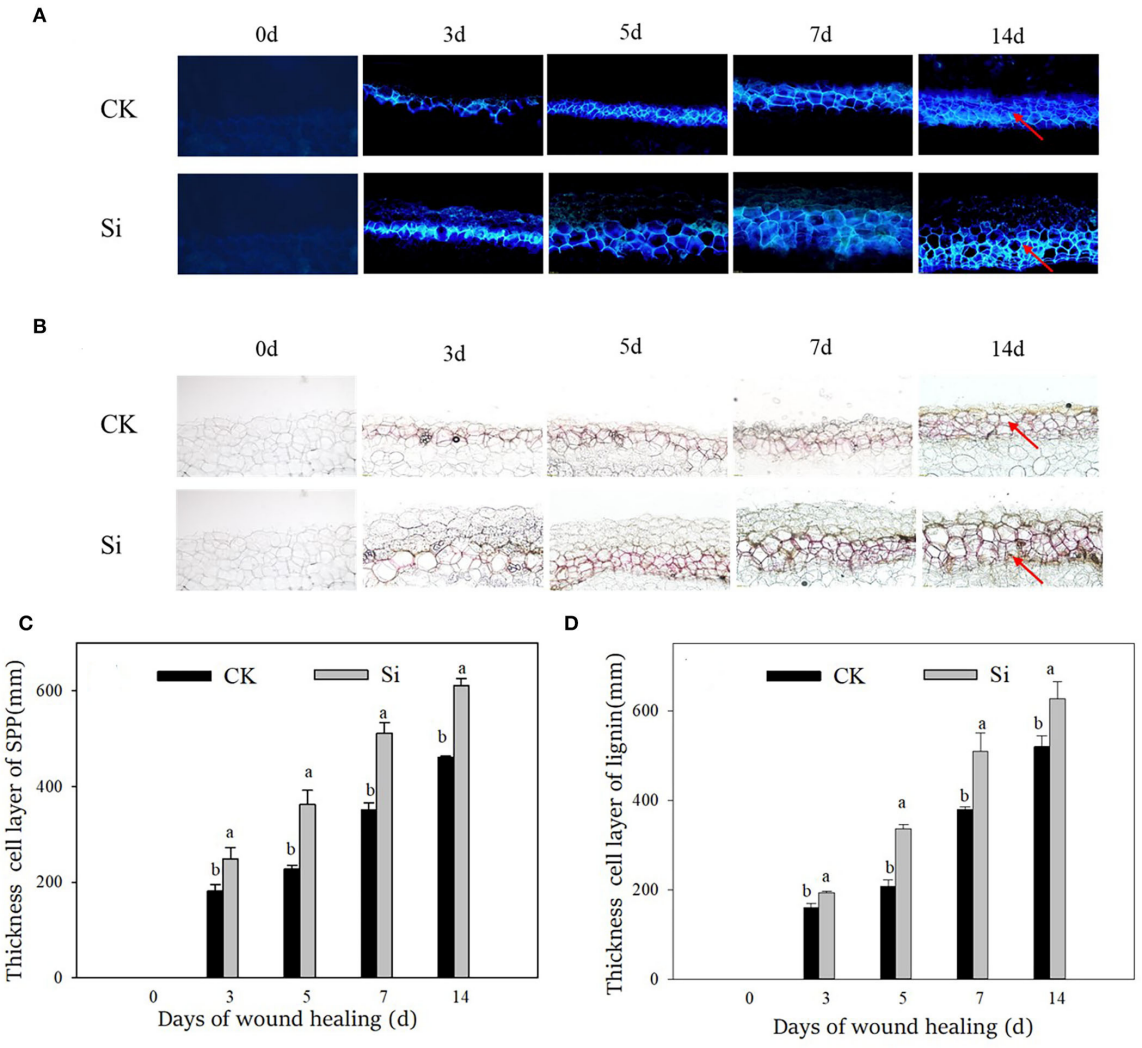
Effect of sodium silicate treatment on the accumulation and cell layer thickness of SPP and lignin during healing. **(A)** Accumulation of SPP. **(B)** Accumulation of lignin. **(C)** Cell layer thickness of SPP. **(D)** Cell layer thickness of lignin. Red arrows represent the accumulation site of SPP and lignin. Vertical bars indicate the standard error of three replicate assays. Columns with different letters at each time point are significantly different (LSD, *P* < 0.05).

Both SPP and lignin increased gradually during wound healing through microscopic observation (Figures 2A,B), corresponding with the increasing thickness of the cell layer (Figures 2C,D). The deposition of SPP and lignin and their cell layer thickness in the Si group were always higher than those in the CK group. Specifically, the cell layer thickness of SPP and lignin in the Si group was 46% and 34% higher than that in the CK group on day 7, respectively.

### Sodium silicate treatment induced the expression levels and enzyme activity of PAL, 4CL, and CAD

**Figure 3:**
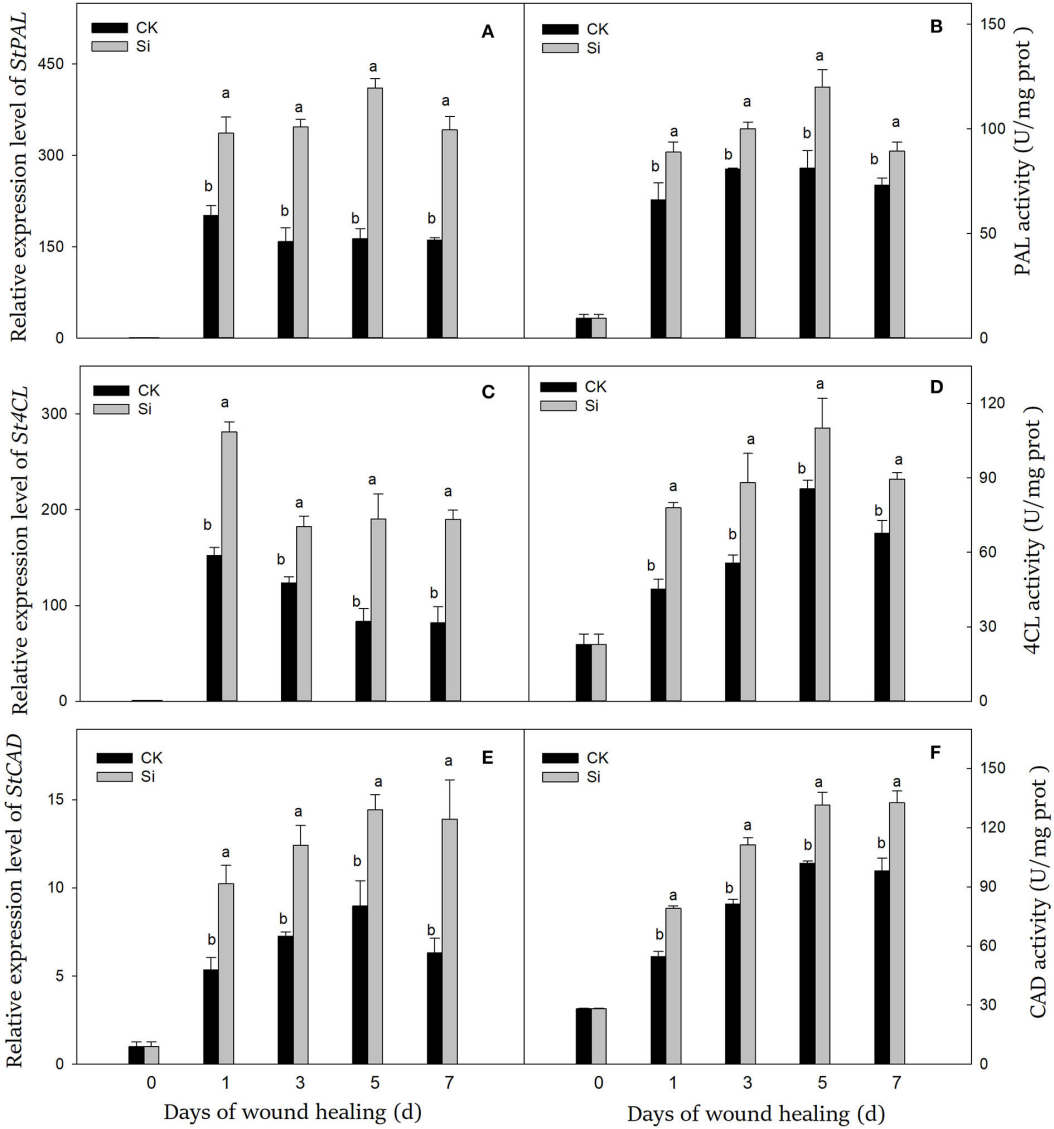
Effect of sodium silicate treatment on the expression of key genes and related enzyme activity of phenylpropane metabolism. The expression levels of *StPAL* **(A)**, *St4CL* **(C)**, and *StCAD* **(E)** genes. The activity of PAL **(B)**, 4CL **(D)**, and CAD **(F)** enzymes. Vertical bars indicate the standard error of three replicate assays. Columns with different letters at each time point are significantly different (LSD, *P* < 0.05).

After the tubers were wounded, the genes expression level and the enzyme activity of PAL, 4CL, and CAD were significantly induced (Figure 3). At the early stage, the expression levels of *StPAL, St4CL*, and *StCAD* were 202, 152, and 5.3 times higher than that on day 0, and the enzyme activities were 6.9, 2.0, and 1.9 times higher, respectively. The expression of *StPAL* and PAL activity remained at high levels during healing. The expression level and enzyme activity of PAL in the Si group were always higher than those in the CK group, which were 113% and 22% higher than CK on day 7, respectively (Figures 3A,B). Similarly, sodium silicate dramatically induced the gene expression of *St4CL* and 4CL activity (Figures 3C,D). The gene expression and enzyme activity of 4CL in the Si group were 132% and 33% higher than CK on day 7, respectively. In addition, the expression level of *StCAD* gene and CAD enzyme activity increased gradually (Figures 3E,F). Both of them in the Si group were higher than those in the CK group, which were 120% and 35% higher than CK on day 7, respectively.

### Sodium silicate treatment induced the synthesis of lignin precursors and lignin

**Figure 4:**
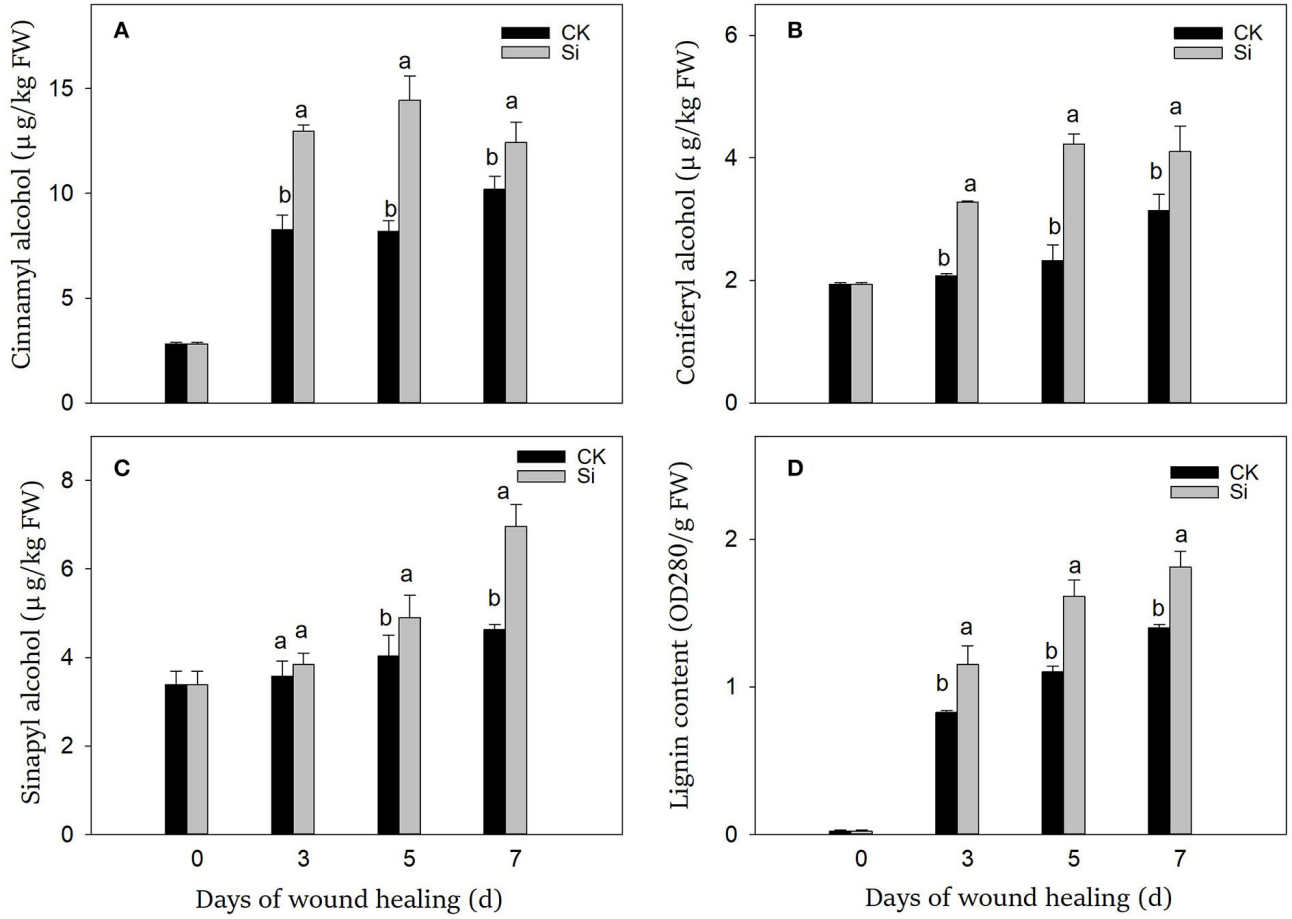
Effect of sodium silicate treatment on the contents of lignin precursors and lignin. The contents of cinnamyl alcohol **(A)**, coniferyl alcohol **(B)**, sinapyl alcohol **(C)**, and lignin **(D)**. Vertical bars indicate the standard error of three replicate assays. Columns with different letters at each time point are significantly different (LSD, *P* < 0.05).

During wound healing, the contents of lignin precursors and lignin increased significantly (Figure 4). During the whole healing process, the accumulation of cinnamyl alcohol, coniferyl alcohol, and lignin was dramatically promoted by sodium silicate treatment (Figures 4A,B,D). Meanwhile, sodium silicate also promoted the accumulation of sinapyl alcohol in the middle and late stages of healing (Figure 4C). Specifically, the contents of cinnamyl alcohol, coniferyl alcohol, sinapyl alcohol, and lignin in the Si group were 22%, 31%, 50%, and 29% higher than those in the CK group on day 7, respectively (*P* < 0.05).

### Sodium silicate treatment promoted the expression levels of *StPOD, StNOX*, and the production of ROS

**Figure 5:**
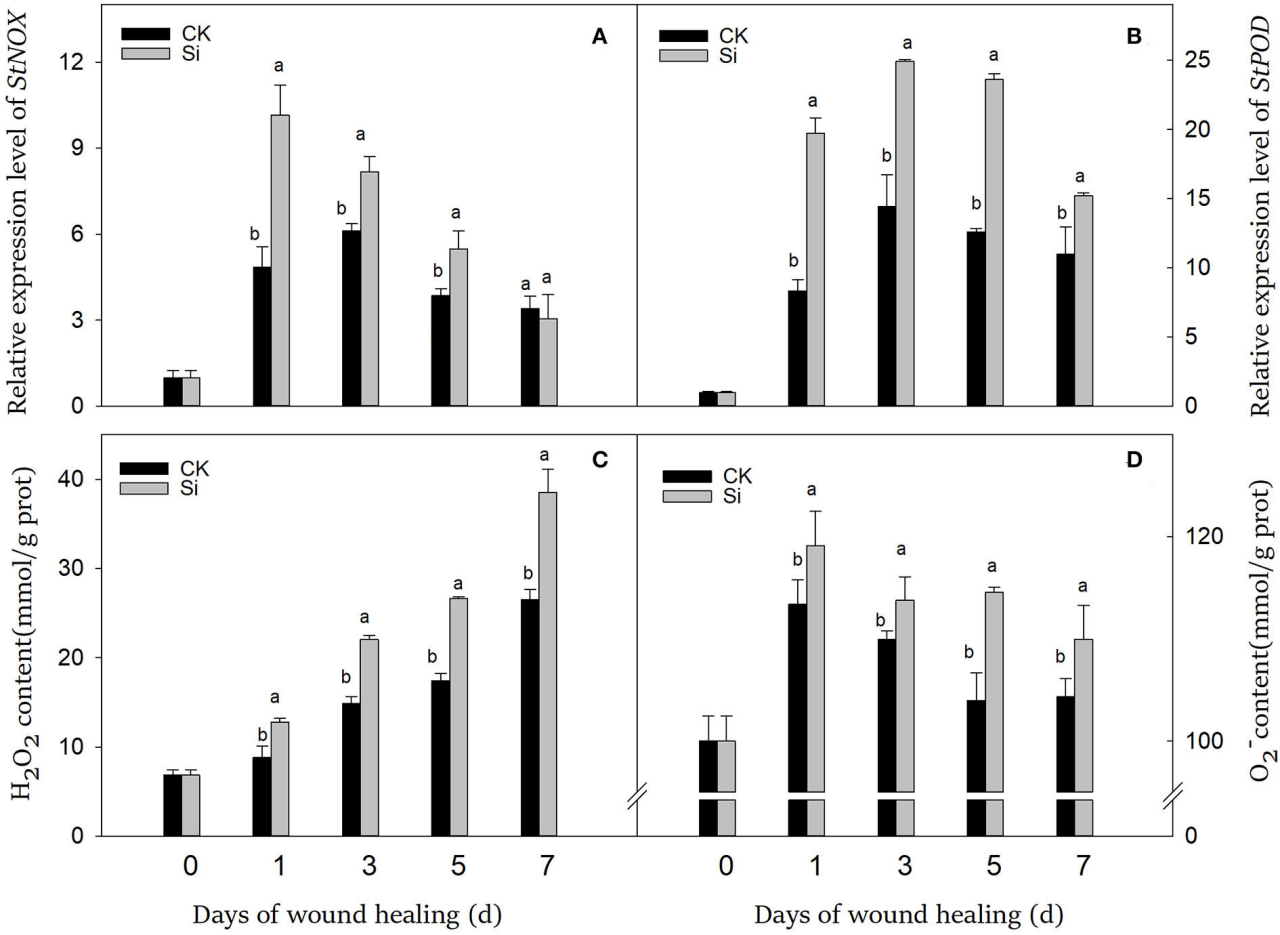
Effect of sodium silicate treatment on the expression of *StNOX* and *StPOD* genes and the contents of ROS. The expression levels of *StNOX* **(A)** and *StPOD* **(B)** genes. The contents of ROS, including H_2_O_2_ **(C)** and ${\text{O}}_{2}^{-}$ **(D)**. Vertical bars indicate the standard error of three replicate assays. Columns with different letters at each time point are significantly different (LSD, *P* < 0.05).

During wound healing, the expression of *StPOD* and *StNOX* showed a trend of first ascending and then descending (Figures 5A,B). Especially in the early and middle stages, sodium silicate dramatically promoted the expression of *StPOD* and *StNOX*. During wound healing, the accumulation of H_2_O_2_ increased gradually, while the accumulation of ${\text{O}}_{2}^{-}$ remained at a higher level (Figures 5C,D). The contents of H_2_O_2_ and ${\text{O}}_{2}^{-}$ in the Si group were always higher than that in the CK group, which were 45% and 5% higher on day 7, respectively (*P* < 0.05).

### Sodium silicate treatment promoted the production of antimicrobial components

**Figure 6:**
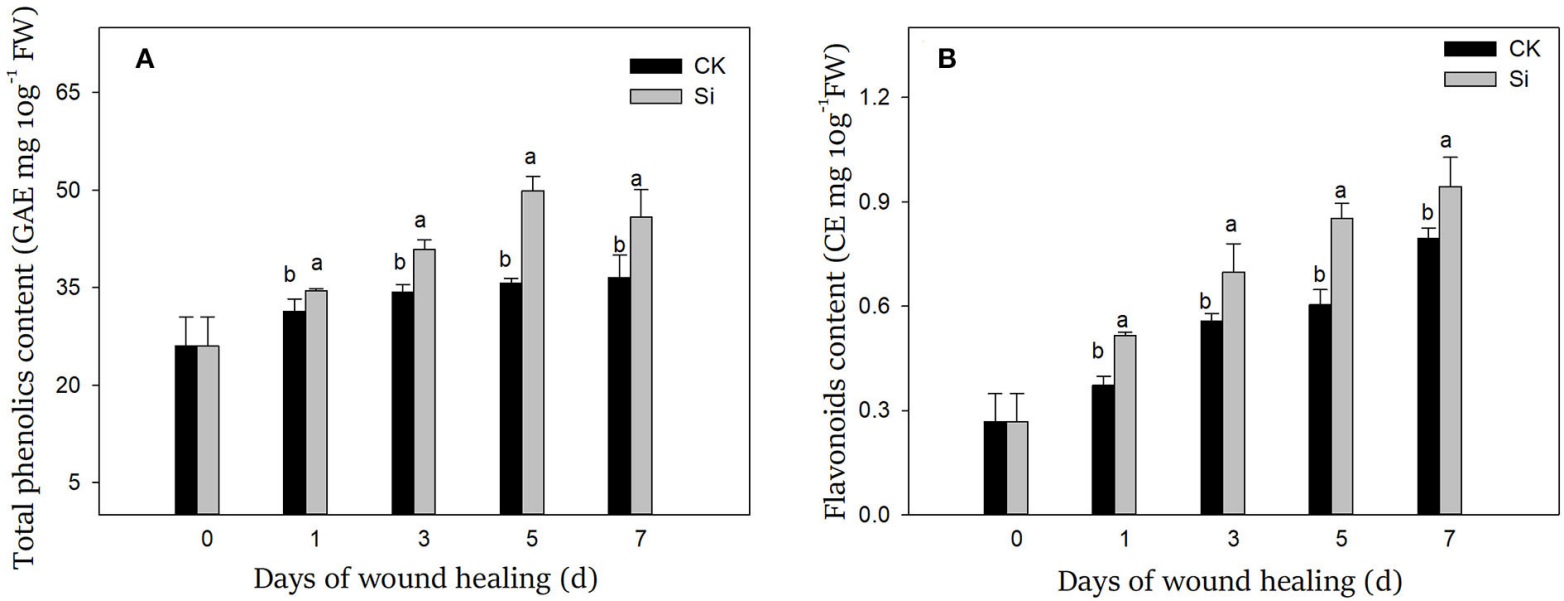
Effect of sodium silicate treatment on the contents of total phenolics **(A)** and flavonoids **(B)**. Vertical bars indicate the standard error of three replicate assays. Columns with different letters at each time point are significantly different (LSD, *P* < 0.05).

During wound healing, the contents of total phenolics and flavonoids increased gradually. Total phenolics and flavonoids in the Si group were always higher than that in the CK group (Figure 6). On day 7, both total phenolics and flavonoids in the Si group were 25% and 18% higher than that in the CK group, respectively.

## Discussion

In essence, the deposition of macromolecules, such as suberin and lignin, at wound sites signals the healing of wounds of tubers (Sabba and Lulai, 2002; Lulai and Neubauer, 2014). The disease index and weight loss rate are important indexes to evaluate the effect of wound healing (Li et al., 2021). In our study, sodium silicate treatment dramatically reduced the weight loss rate and disease index. More interestingly, the deposition of a white healing structure was apparent in the tubers treated with sodium silicate (Fig 1). Furthermore, the deposition of SPP and lignin in the Si group was higher than that of the CK group (Fig 2). Si can deposit directly in plant cells to enhance their barrier function. For example, Si could deposit in the leaf epidermis by spraying silicon fertilizer on the leaf surface, thus improving the resistance of rice to blast disease (Abed-Ashtiani et al., 2012). The application of 5.0% calcium silicate on leaves not only increased the activity of polyphenol oxidase in peanut leaves but also made the largest deposition of Si in plants (Packirisamy et al., 2019). Therefore, we suggest that Si can not only form deposits directly at the wound site but also induce the accumulation of typical healing structures such as SPP and lignin. This physical barrier at the wound site could strengthen cell protection and prevent water evaporation.

Phenylpropane metabolism played an important role in wound healing, which provided necessary substrates for the accumulation of SPP and lignin (Woolfson et al., 2018). PAL catalyzed the formation of cinnamic acid from L-phenylalanine, which was the first step of phenylpropane metabolism (Kumar and Knowles, 2003). Cinnamic acid was then converted into other phenolic acids and further converted into phenolic acid-CoA in the presence of 4CL (Li et al., 2021). These substances could participate in the formation of SPP or further generate lignin precursors under the catalysis of CAD (Capote et al., 2018). In our study, sodium silicate could dramatically activate the gene expression levels and enzyme activities of PAL, 4CL, and CAD during wound healing (Figure 3). Lignin was an important component of healing structure, which was synthesized by the polymerization of three kinds of lignin precursors, namely, coniferyl alcohol, sinapyl alcohol, and cinnamyl alcohol (Zheng et al., 2020). Through further testing, the contents of the three kinds of lignin precursors and lignin in the Si group were found to be significantly induced (Figure 4). Similar reports showed that sodium silicate can promote the expression levels of *PAL, C4H*, and *4CL* genes in maize, and the contents of chlorogenic acid and total phenolics were higher than the control (Wang et al., 2021). Si enhanced the expression level of *PAL* in oat and increased the degree of cell lignification (Asgari et al., 2018). Si could also improve the resistance of wheat leaves to tan spot, and the degree of disease reduction was related to the activation intensity of phenylpropane metabolism (Dorneles et al., 2018). The application of Si alleviated cold stress in plants by increasing ABA and JA synthesis (Moradtalab et al., 2018). Under salt stress, the addition of Si could trigger ABA signaling by activating the enzyme related to ABA synthesis (Al Murad et al., 2020). Similarly, Si treatment could also enhance the expression of JA synthesis-related genes under injury stress (Tripathi et al., 2021). ABA and JA were widely reported to accelerate the wound healing by promoting phenylpropanoid metabolism in potato, tomato, and kiwi fruit (Leide et al., 2012; Zhou et al., 2019; Wei et al., 2020). Therefore, we hypothesized that Si might activate the phenylpropane metabolism by stimulating intracellular hormone signals such as ABA and JA, thus promoting the synthesis of substrates of SPP and lignin in tubers.

Once tubers were damaged, a large number of ${\text{O}}_{2}^{-}$ could be produced, which was mainly catalyzed by NOX (Kumar et al., 2007). Unstable ${\text{O}}_{2}^{-}$ could be further transformed into H_2_O_2_, which were the main ROS components in the healing process (Mohanta et al., 2018). Generally, two oxygen bursts occur after mechanical injury of tubers. For the first time, a small amount of ROS could be used as signal molecules to activate the defense response at the initial stage of healing (Liu and He, 2017). Second, in the middle and late periods after tuber injury, a large number of ROS participated in oxidative crosslinking of healing structures (Denness et al., 2011). Similar to H_2_O_2_, POD could take part in the cross-linking of suberin and lignin (Vishwanath et al., 2015). Thus, reactive oxygen metabolism was involved not only in healing signaling but also in oxidative crosslinking of suberin and lignin (Kumar et al., 2007). In our study, sodium silicate treatment could promote the expression of *StPOD* and *StNOX* and the accumulation of ${\text{O}}_{2}^{-}$ and H_2_O_2_ (Figure 5)_._ Similarly, Si treatment can induce the generation of ROS in potato, thus enhancing the resistance of leaves to late blight (Xue et al., 2021). Sodium silicate treatment can also enhance the accumulation of H_2_O_2_ and ${\text{O}}_{2}^{-}$ in melon fruits and improve the resistance to *Trichothecium roseum* (Lyu et al., 2019). The combined treatment of Si and chitosan increased the content of ${\text{O}}_{2}^{-}$ and H_2_O_2_ in jujube fruits and induced the resistance of fruits to Alternaria rot (Guo et al., 2019). Si activated POD activity in the tomato plant and enhanced its resistance to early blight (Gulzar et al., 2021). Sodium silicate treatment could promote the contents of total phenolics and lignin in ryegrass, which was associated with increased POD activity (Ribera-Fonseca et al., 2018). Therefore, we suggest that Si could induce the generation of ROS by activating NOX. On the one hand, ROS was involved in the transmission of wound stress signal, and on the other hand, it could participate in oxidative crosslinking of suberin monomers and lignin precursors with POD.

During the wound healing of tubers, a large number of antimicrobial substances, such as total phenolics and flavonoids, can be produced at the wound site. In addition to participating in the production of SPP and lignin, phenolics can also inhibit bacteria directly or form quinones toxic to pathogens by the oxidation of polyphenol oxidase (PPO) (Shadle et al., 2003). Flavonoids function to scavenge free radicals and inhibit fungal bud tube elongation and mycelium growth (Gao et al., 2018). In our study, sodium silicate could promote the synthesis of total phenolics and flavonoids during healing (Figure 6). Similar reports showed that Si increased the content of phenolics in wheat, which had fungitoxic effects (Dorneles et al., 2018). Low concentration of Si could increase the synthesis of total phenolics, monophenols, and flavonoids in buckwheat (Azad et al., 2021). Si promoted the content of phenolics and flavonoids in strawberry and enhanced the resistance of plants to salt stress (Yaghubi et al., 2019). Si also promoted the synthesis of phenolics in the avocado peel, thus improving the postharvest quality of avocado fruit (Tesfay et al., 2011). Furthermore, Si could directly inhibit the growth of mycelia of many types of fungi *in vitro* (Bi et al., 2006). Therefore, we speculated that Si could not only directly inhibit pathogenic bacteria but also promote the accumulation of antimicrobial substances such as total phenolics and flavonoids by enhancing secondary metabolism.

## Conclusion

Sodium silicate induced the expression levels and enzyme activity of PAL, 4CL, and CAD in phenylpropane metabolism, which promoted the synthesis of substrates of SPP and lignin, such as coniferyl alcohol, cinnamyl alcohol, and sinapyl alcohol. Meanwhile, NOX and POD were induced, and the content of O^2−^ and H_2_O_2_ was promoted, which could be used for injury signal transmission and oxidative crosslinking of SPP monomers and lignin precursors. Finally, sodium silicate treatment enhanced the deposition of antimicrobial compounds, SPP, and lignin at the wound site and effectively reduced the weight loss and disease index of tubers during healing.

## Data availability statement

The original contributions presented in the study are included in the article/supplementary material, further inquiries can be directed to the corresponding author.

## Author contributions

Designed research, formal analysis, writing, and review: YH. Data curation: RY and QW. Resources: BW and DP. All authors contributed to the article and approved the submitted version.

## Funding

This work was supported by the Research Program Sponsored by the State Key Laboratory of Aridland Crop Science (No. GSCS-2021-06).

## Conflict of interest

The authors declare that the research was conducted in the absence of any commercial or financial relationships that could be construed as a potential conflict of interest.

## Publisher's note

All claims expressed in this article are solely those of the authors and do not necessarily represent those of their affiliated organizations, or those of the publisher, the editors and the reviewers. Any product that may be evaluated in this article, or claim that may be made by its manufacturer, is not guaranteed or endorsed by the publisher.
